# Internet-Delivered Therapy for Parents With Health Anxiety by Proxy: Protocol for a Single-Case Experimental Design Study

**DOI:** 10.2196/46927

**Published:** 2023-11-24

**Authors:** Katrine Ingeman, Lisbeth Frostholm, Kaare Bro Wellnitz, Kristi Wright, Ditte Hoffmann Frydendal, Patrick Onghena, Charlotte Ulrikka Rask

**Affiliations:** 1 Research Unit Department of Child and Adolescent Psychiatry Aarhus University Hospital Psychiatry Aarhus Denmark; 2 Department of Clinical Medicine Aarhus University Aarhus Denmark; 3 The Research Clinic for Functional Disorders and Psychosomatics Aarhus University Hospital Aarhus Denmark; 4 Department of Psychology Faculty of Arts University of Regina Regina, SK Canada; 5 Faculty of Psychology and Educational Sciences KU Leuven Leuven Belgium

**Keywords:** health anxiety by proxy, health anxiety, internet-delivered treatment, single-case experimental design, SCED, protocol

## Abstract

**Background:**

Health anxiety (HA) by proxy is described as parents’ obsessive worries that their child is severely ill although this is not medically confirmed. Research on HA by proxy suggests that it is highly distressing for the parent and that the child may be at risk of developing maladaptive symptom coping strategies. No targeted treatment for this group exists. We developed PROXY, an 8-week psychological internet-delivered treatment for parents with HA by proxy. The treatment components of PROXY are informed by cognitive behavioral therapy as well as acceptance and commitment therapy, and it was developed in collaboration with parents experiencing HA by proxy and clinical experts.

**Objective:**

This paper describes the protocol for a study investigating the potential effects of PROXY on parents’ worries about their children’s health using a single-case experimental design (SCED).

**Methods:**

Five parents clinically evaluated as experiencing HA by proxy will be included. A replicated randomized SCED study will be conducted in which each participant will be randomized to receive treatment after a baseline period of between 7 and 26 days (phase A). The treatment phase duration is 8 weeks for all participants (phase B), followed by a follow-up phase lasting between 14 and 33 days (phase C), ensuring that all participants remain in the study for 96 days. Participants will report daily anxiety level by SMS text message throughout the study. They will also answer self-report questionnaires, including questions on HA by proxy and own HA, 4 times during the study. Data will be submitted to structured visual analysis to inspect anxiety level, the variability of anxiety, trends, the overlap of data points among phases, when effects occur, and the consistency of data patterns across participants. Furthermore, randomization tests will be conducted for each participant to test the null hypothesis that PROXY will have no effect on participants’ anxiety.

**Results:**

The recruitment of parents began in June 2022. As of March 2, 2023, a total of 4 parents have been included in the study. Data collection was expected to cease in April 2023.

**Conclusions:**

To the best of our knowledge, this protocol describes the only study of treatment for HA by proxy. As the prevalence of this condition is still unknown, a SCED was chosen because this method allows the inclusion of very few participants while still providing information on effects and treatment courses. Conducting the study with a replicated randomized phase design enables methodologically sound testing despite the inclusion of very few participants. The results will inform researchers on individual treatment courses and effects, which may direct future research in terms of the possible mechanisms of change, ideas for how to refine the treatment content, and how the treatment may be offered to patients in the future.

**Trial Registration:**

ClinicalTrials.gov NCT04830605; https://clinicaltrials.gov/study/NCT04830605

**International Registered Report Identifier (IRRID):**

DERR1-10.2196/46927

## Introduction

### Background

Health anxiety (HA) by proxy is defined as parents’ excessive fear that their child is experiencing a serious illness although this is not confirmed by a medical professional [1]. It is characterized by obsessive rumination about the health of one’s child and is often accompanied by control or avoidance behavior, such as facilitating repeated medical evaluations of the child or avoiding all information about child illnesses [2]. More severe cases may be observed in parents diagnosed with HA [3], but milder cases on the continuum are also likely to be recognized by health professionals working in pediatric settings.

A recent qualitative study investigating the lived experiences of parents with HA by proxy found that these parents experience significant distress as a result of their anxiety, and they struggle with finding the right balance in worrying about their child’s health [4]. Furthermore, the parent’s relationships with their child, other parent, and health professionals are affected [4].

HA by proxy may also have consequences for the child. Evidence suggests that HA symptoms in parents with health worries and increased focus on somatic concerns play a significant role in HA symptoms in children [5]. Furthermore, the parent’s response to the child’s health concerns [6] and how the parent copes with their own symptoms have been found to influence the health attitudes and behaviors of the child [7-10]. This risk may be especially high in HA by proxy because the child is exposed to a particular parental preoccupation with symptoms and fear of illness.

The members of the research team behind this study have previously investigated the assessment of HA by proxy by developing the Health Anxiety by Proxy Scale (HAPYS) with promising results [2]. Now that parents with excessive worries over their child’s health can be identified, the next important step is to develop effective treatment. Existing treatments do not specifically address HA by proxy. A treatment program has already been developed to target parents with anxiety to prevent anxiety in their offspring [11], and effective psychological treatments for HA also exist in both face-to-face and internet-delivered formats [12-14]. Specifically, a recently developed treatment program for HA based on the principles of acceptance and commitment therapy (ACT) and delivered over the internet (iACT) [15] has shown promising results [14,16] and was usable as a template for the development of specialized treatment for parents with HA by proxy.

### Developing an Internet-Delivered Treatment Program for HA by Proxy (PROXY)

PROXY is inspired by the existing iACT program for HA and uses the same web platform [17]. The treatment content for PROXY was developed de novo in several steps by 4 of the authors: 3 psychologists (KI, DHF, and LF) and a child and adolescent psychiatrist (CUR) with clinical and research experience with HA. Furthermore, 2 of these authors (LF and CUR) are also trained specialists in cognitive behavioral therapy (CBT). The development process included a number of steps. First, ideas for content, themes, exercises, videos, and audio files were brainstormed. These were presented as sketches to 3 parents diagnosed with HA and with worries about their children’s health who subsequently provided group feedback.

Next, the treatment manual went through several rounds of feedback from the project group. Further written feedback on the text manual was provided by 2 (67%) of the aforementioned 3 parents with individual comments in Microsoft Word document format. These 2 parents also participated in patient videos about living with HA by proxy, how to involve relatives, and how to speak to their children about their anxiety. Finally, the content was programmed on a web platform with close collaboration between the project group and web developers.

### Usability Test

The thinking-aloud method was used to test the usability of specific selected parts of PROXY. The method involved participants verbalizing their thoughts *while* using the web-based program [18,19]. A male layperson aged 25 years without prior knowledge about the treatment and a female patient aged 47 years with HA by proxy and prior ACT group treatment of HA explored how they experienced (1) navigating the treatment and (2) potential technical issues, as well as (3) how they understood the program. Generally, they navigated the platform easily. Minor adjustments to the instructions, navigation, the choice of wording, and models were made.

### Final Version of PROXY

PROXY is an 8-week therapist-supported internet-delivered program containing written psychoeducation, audio files, behavioral exposure exercises, homework, and videos distributed in 8 modules (Textbox 1). The therapist answers questions and provides feedback every week using an embedded secure message system.

**Overview of module content.**

**Week 1**

- Gain knowledge about health anxiety (HA) by proxy (module 1)
  Helper: knowledge
  Aim: for the parent to obtain knowledge about having HA on behalf of their child and explore how they can begin to understand their own anxiety
  Exercises: (1) write own story of anxiety and (2) fill in the anxiety spiral
  Videos and audio: (1) parent videos about having HA by proxy, (2) psychoeducation about HA by proxy, and (3) introduction video to the treatment
  Homework: pay attention to when and where anxiety arises

- Learn about values (module 2)
  Helper: values
  Aim: to help the parent identify their own values as a parent and motivate them to start coping differently with their anxiety
  Exercises: (1) consequences of the anxiety, (2) reflections on being a good parent, and (3) own values as a parent
  Videos and audio: audio with focus on identifying values
  Homework: practice putting off worries and set aside a specific time of day to worry

**Week 2**

- Look at the anxiety from the outside (module 3)
  Helper: defusion
  Aim: introduction to anxiety-related control and avoidance strategies, followed by exercise to investigate own behaviors; learning how to defuse from thoughts and feelings; the process of coping differently with the anxiety is initiated
  Exercises: (1) “Don’t think about the ice cream,” (2) behavior analysis, and (3) defusion exercise
  Videos and audio: audio with defusion exercise
  Homework: practice putting off control and avoidance behavior

**Week 3**

- Practice acceptance (module 4)
  Helper: acceptance
  Aim: to practice accepting and holding the anxiety as a new way of coping
  Exercises: (1) stop, breathe, observe inner states, and prioritize
  Videos and audio: (1) audio with acceptance exercise and (2) audio with body scan
  Homework: practice having more breaks during the day, and practice acceptance with mindfulness audio files

**Week 4**

- Challenge the anxiety (module 5)
  Helper: exposure
  Aim: to motivate parents to gradually expose themselves to their anxiety; focus on letting personal values direct choices instead of control and avoidance strategies
  Exercises: (1) exposure exercise with pictures of sick children, (2) drafting their anxiety hierarchy, and (3) exposure work sheet
  Videos and audio: none
  Homework: practice exposure
  Extra: a module for relatives is introduced with the aim of providing information on HA by proxy and how to be supportive during treatment, including exposure exercises

**Week 5**

- Practice self-compassion (module 6)
  Helper: self-compassion
  Aim: show the connection among one’s expectations, stress, and anxiety, along with how one can be more kind to oneself and practice self-compassion
  Exercises: (1) “How would you treat a friend?” (2) working with own conditional assumptions
  Videos and audio: audio on self-compassion
  Homework: do something nice for oneself and practice exposure

**Week 6**

- Use your network (module 7)
  Helper: parents’ network
  Aim: to help parents use their close network for support and provide tools to talk about their anxiety with others, including their child
  Exercises: (1) How is the anxiety affecting own close relations? (2) talk about the anxiety with a friend and partner, and (3) talk about the anxiety with child and or write a letter to child
  Videos and audio: (1) parent videos about talking to their children about their anxiety and (2) parent video about family support
  Homework: talk to child about anxiety and practice exposure

**Weeks 7 and 8**

- Your path further (module 8)
  Aim: to recap the treatment content, emphasize why it is important to continue working with acceptance, values and exposure, and how to prevent relapse; furthermore, advice about how to handle future child symptoms and physician visits is provided
  Exercises: (1) continued work and insights from the treatment and (2) strategies to apply in the event of setbacks
  Videos and audio: none

The content framework is based on a combination of ACT [15] and CBT [20]. ACT aims to create greater psychological flexibility that allows (1) full awareness of here-and-now experiences, with an attitude of openness and curiosity; and (2) conscious and deliberate decisions inspired by core values, that is, deepest desires for who one wants to be and what one wants to stand for in life [15,21]. Thus, PROXY targets parents’ maladaptive anxiety-driven behaviors toward their child with focus on their gradual exposure to the things or situations that trigger their anxiety. This means that the CBT concept of exposure is urged to be based on the patient’s personal value. Furthermore, practicing acceptance helps to reduce the influence and impact of the painful thoughts and feelings that arise during the exposure.

Each of the first 7 modules in PROXY offers a *helper*, which is a treatment component (eg, awareness of values, acceptance, and self-compassion) that will help the patient move in a value-based direction in life instead of being trapped trying to control and avoid anxiety (Textbox 1).

### Aims

In patient groups in which the prevalence of a health condition is low or unknown, as is the case with HA by proxy, randomized controlled trials are either not possible or unlikely to succeed [22]. Single-case experimental design (SCED) studies are used in clinical psychology to determine whether an effect of an intervention at an individual level has occurred [23-25]. Thus, a SCED study allows testing the effect of treatment for parents with HA by proxy in a small sample size while still controlling for external factors and upholding internal validity [26].

Consequently, this paper aims to describe the design for the first testing of PROXY using a SCED. The study investigates the potential effect of PROXY on parents’ worries about their children’s health. We hypothesize an initial increase in worries and anxiety, followed by a delayed and gradual reduction.

## Methods

### Study Design

A SCED study implies control of the independent variable (here, PROXY) while measuring the dependent variable (here, parental worries about their child’s health) repetitively and often [24]. SCEDs should be distinguished from both case studies, which are not experimental but descriptive, and designs based on group comparisons where the experimental unit is the participant and where participants are assigned to different groups [27]. In a SCED, the experimental units are the repeated measures of a specific variable under investigation. Various types of SCEDs exist, but only the replicated randomized single-case AB phase design type will be elaborated here [23,27].

The project was registered on ClinicalTrials.gov on April 2, 2021 (NCT04830605).

### A Replicated Randomized Single-Case ABC Phase Design

This study will apply a replicated randomized single-case AB phase design for testing the treatment effects. AB designs are particularly useful when testing a psychological treatment that cannot be subjected to withdrawal or to repeated reversal between treatment and baseline. By adding a randomization feature and a replication feature to this basic design, both the internal and external validity are strengthened [27-29]; for example, potential confounding variables related to time, such as natural changes in the participants’ anxiety level or events that may affect anxiety, are statistically controlled for when the time point for the introduction of the intervention is randomized. In addition, replicating the design adds to the generalizability.

**Figure 1:**
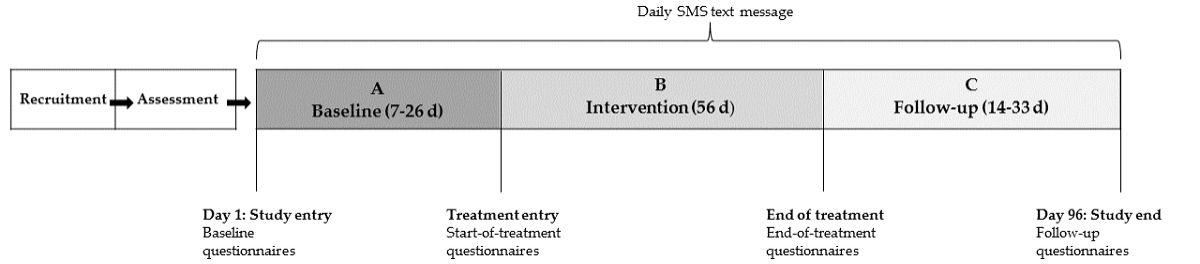
Study flowchart for the single-case experimental design (refer to Table 1 for specifics on questionnaires).

Specifically for this study, the intervention is randomly introduced after 7 to 26 days in the baseline phase (phase A). After the intervention (phase B), a follow-up phase (phase C) lasting between 14 and 33 days, depending on when the intervention was introduced, ensures that every participant has the same number of daily measurement points throughout the study (96 in total; Figure 1).

**Table 1:** Overview of outcome measures, instruments, and distribution time points.

Outcome			Instrument		Time point												
					D^a^		W^b^		Re^c^		BL^d^		Start^e^		EoT^f^		FU^g^
**Primary outcome**																	
	Symptoms and effect on daily life of health anxiety by proxy	SMS text message		✓													
**Secondary outcomes**																	
	Health anxiety by proxy	HAPYS^h^						✓		✓		✓		✓		✓	
	Illness behavior when a child has symptoms	ARCS^i^						✓		✓		✓		✓		✓	
	Catastrophic thinking about child’s symptoms	PCS-P^j^						✓		✓		✓		✓		✓	
	Illness worry	WI-6-R^k^						✓		✓		✓		✓		✓	
	Depression and anxiety	SCL-12^l^						✓		✓		✓		✓		✓	
	General well-being	WHO-5^m^						✓		✓		✓		✓		✓	
**Feasibility outcomes**																	
	Experience of treatment	ESQ^n^												✓			
	Negative effects of treatment	NEQ-20^o^												✓			
	Experience of internet-delivered treatment	IEUQ^p^												✓			
	SMS text message evaluation	Selected items														✓	
	Feasibility measures	Free writing				✓								✓			
**Other**																	
	Suicide risk	Single item from the SCL^q^				✓											
	Bodily symptoms	BDS^r^ checklist						✓									

^a^D: daily.

^b^W: weekly.

^c^Re: questionnaire filled in at self-referral.

^d^BL: baseline.

^e^Start: start of treatment.

^f^EoT: end of treatment.

^g^FU: follow-up after 96 days from study entry.

^h^HAPYS: Health Anxiety by Proxy Scale.

^i^ARCS: Adult Response to Children’s Symptoms.

^j^PCS-P: Pain Catastrophizing Scale–Parent version.

^k^WI-6-R: Whiteley Index-6 Revised.

^l^SCL-12: Symptom Checklist-12.

^m^WHO-5: World Health Organization-5 Well-Being Index.

^n^ESQ: Experience of Service Questionnaire.

^o^NEQ-20: Negative Effects Questionnaire-20.

^p^IEUQ: Internet Evaluation and Utility Questionnaire.

^q^“Thoughts of ending your life.”

^r^BDS: bodily distress syndrome.

### Participants

Five parents assessed with HA by proxy using the HAPYS [2] will be recruited for the study. No more than 3 participants may be allocated to a baseline duration of between 7 and 10 days to ensure that the participants will have different start times. The specific inclusion and exclusion criteria are presented in Textbox 2.

**Inclusion and exclusion criteria.**

**Inclusion criteria**

- Parents aged >18 y
- Assessed with health anxiety by proxy
- At least 1 child aged <18 y
- Read, write, and speak Danish

**Exclusion criteria**

- Comorbid diagnoses of substance abuse, bipolar disorder, psychotic disorders (International Classification of Diseases, Tenth Revision [ICD-10], codes: F20-29), or autism spectrum disorder
- Suicidal risk
- Recently started taking psychotropic drug therapy (within the last 2 mo)
- Child with severe health problems requiring care in hospital setting

### Recruitment

Participants are recruited in two ways: (1) participants self-refer to the project through the secure email system at the Research Clinic for Functional Disorders and Psychosomatics (hereinafter the Research Clinic), Aarhus University Hospital, and complete the project questionnaire (Table 1) in Research Electronic Data Capture (REDCap; Vanderbilt University), which includes written consent to assessment [30,31], and, after a diagnostic interview, they are included in the project, if eligible; and (2) participants are recruited at their ordinary assessment for HA at the Research Clinic if they are also clinically assessed as experiencing excessive worries about their child’s health.

### Assessment

Participants are assessed using a short standardized diagnostic interview based on schedules for clinical assessment in neuropsychiatry (SCAN) [32,33] and supplemented with an assessment of HA by proxy using the HAPYS.

### Ethical Considerations

All participants receive written and verbal information about the project before signing the consent to participate. The project has been approved by the Danish research ethics committee (1-10-72-296-20) and registered with the Danish data protection agency (1-16-02-921-17).

### Outcome Measures

#### Primary Outcome

The primary outcome measures of HA by proxy are answered through a link in an SMS text message sent to the participants every day during the study period. The measures contain 3 items selected from the HAPYS [2] (1-3), 1 impact questionnaire (4), and 1 de novo formulated item on committed action (5). All items are answered on a scale ranging from 1 to 10 (Textbox 3; coded as 0-9 for analyses). A daily score is summed for items 1 to 3 (range 0-27), and items 4 and 5 are assessed individually (range 0-9). The 3 items from the HAPYS were selected by the project group based on the following considerations: items that have excellent face validity as evaluated by clinical experts (2 psychologists and 1 child and adolescent psychiatrist) and items indicative of high sensitivity to change as evaluated by looking at the sensitivity to change on similar items from the Whiteley-7 scale [34] using data from a randomized controlled trial of internet-delivered ACT for HA [14].

**Questions sent via SMS text message.**

**“On a scale ranging from 1 to 10, how well do the following statements describe your day? 1=‘does not describe it at all’ and 10=‘describes it very well.’”**

- “I have had persistent worries about my child’s health.”
- “I have been worried that my child suffers from a serious physical illness.”
- “I have had the need to reassure myself by seeking a physician, examining my child, Googling symptoms, or something else.”
- “My anxiety for my child’s health has influenced my time together with my child.”
- “I have done something in the past 24 hours that was important to me in spite of my anxiety.”

#### Secondary Outcomes

The secondary outcome measures are full questionnaires answered 4 times during the study period. An overview of measures and data collection is presented in Table 1.

#### HAPYS Questionnaire

The HAPYS is a 26-item self-report questionnaire assessing HA by proxy, including an impact section with an additional 6 items [2]. The items are rated on a 5-point scale (0=not at all or never, 1=a little or rarely, 2=some or sometimes, 3=quite a lot or often, and 4=a lot or most of the time; range 0-104; higher scores indicate more anxiety), except for the impact section, which is rated on a 4-point scale (0=no; 1=yes, a little bit; 2=yes, quite a bit; and 3=yes, a great deal; range 0-18; higher scores indicate more impact). The HAPYS has shown good psychometric properties [2].

#### Adult Response to Children’s Symptoms–Protect and Monitor Subscales (Revised)

The Adult Response to Children’s Symptoms scale measures parental behavior in relation to the child having abdominal pain and consists of 4 subscales: protect, monitor, minimize, and distract [35]. For this study, a revised Danish version of the protect (13 items) [36] and monitor (4 items) subscales are used, replacing *abdominal pain* with *feels unwell.* The monitor subscale was translated into Danish for this study following World Health Organization (WHO) guidelines [37]. Both scales are measured on a 5-point scale ranging from 0=never to 4=always, averaged to a total score of 0 to 4, with higher scores indicating more protective and monitoring parental behavior. The original scale and the subscales present with satisfactory psychometric properties [38-40].

#### Pain Catastrophizing Scale–Parent Version

The Pain Catastrophizing Scale–Parent version consists of 13 items assessing parents’ thoughts and feelings when their child has pain symptoms (rated on a scale ranging from 0 to 4, with 0=not at all and 4=extremely; range 0-52; higher scores indicate more catastrophizing) [41]. Psychometric investigation has demonstrated a 3-factor model and good internal consistency [41]. The Danish version was translated following WHO guidelines [37] and showed good face validity after cultural adaption [42].

#### Whiteley Index-6 Revised

HA and illness worries are assessed using the Whiteley Index [34,43]. In this study, we use the Whiteley Index-6 Revised, where items concerning somatic symptoms were eliminated, and an item about obsessive illness rumination was included (“Recurrent thoughts about being ill that are difficult to put out of your mind”), resulting in strengthened psychometric properties [44]. The scale consists of 6 items with scores ranging from 0=not at all to 4=a great deal and a summed score of 0 to 24, with higher scores representing higher levels of illness worry.

#### Symptom Checklist

Parents’ levels of distress, anxiety, and depression are screened by the Symptom Checklist-12, which are subscales from the Symptom Checklist-90-Revised [45] that have shown satisfactory sensitivity and specificity in detecting depression and anxiety and general distress in relation to emotional psychiatric disorders [46,47]. The scales are scored from 1=not at all to 5=extremely, and the summed scores are divided by the number of items to yield a mean score (range 1-5).

#### WHO-5 Well-Being Index

The WHO-5 Well-Being Index is a 5-item rating scale measuring subjective well-being on a scale ranging from 0 to 5. The raw score ranging from 0 to 25 is multiplied by 4, providing a final score ranging from 0 to 100, where a lower score represents worse well-being [48]. This scale has demonstrated high validity and is considered a good outcome measure for wanted and unwanted treatment effects [48].

#### Feasibility Outcomes

##### Experience of Service Questionnaire

The Experience of Service Questionnaire measures parents’ experience with the treatment they have received [49,50]. In this study, a modified version will be used where all questions related to physical settings are removed, and 7 questions about impact on the parent, child, and their interaction are added from the Danish revised version developed and used by the Department of Psychology and Behavioral Sciences, Aarhus University [51]. The 14 statements are scored as 0=not true, 1=partly true, and 2=true (range 0-28).

##### Negative Effects Questionnaire-20

The Negative Effects Questionnaire-20 measures 6 factors related to the negative effects of psychological treatment: encompassing symptoms, hopelessness, failure, stigma, dependency, and quality [52]. Each negative effect is rated on a scale ranging from 0=not at all to 4=extremely and attributed to either “The treatment I received” or “Other circumstances.” The 20-item version has demonstrated satisfactory psychometric properties [52].

##### Internet Evaluation and Utility Questionnaire

The Internet Evaluation and Utility Questionnaire will be used to evaluate the experience of receiving internet-delivered treatment. Using 15 items, this instrument measures the usability and utility of an internet-delivered treatment program with questions answered on a 5-point rating scale [53].

##### SMS Text Message Evaluation

Three questions evaluating SMS text message data collection will inquire about how the participants experienced answering an SMS text message every day, whether it had any influence on their daily life, and whether their answers were affected by the fact that they had to do this every day. The questions are answered in free writing and will inform the researchers about the experience of this type of self-monitoring.

##### Evaluation of Modules

After each module, the participants are asked about the content of the modules answered in free writing.

### Analyses

#### Analysis of Primary Outcome Measure

##### Visual Analysis

First, the data collected daily will be submitted to visual analysis, with the scores of the dependent variable on the y-axis and the measurement times on the x-axis. Six main features are visually examined using the guidelines provided in the study by Lane and Gast [54]: anxiety level in the phases, the variability of data points both within and among phases, trends in data, the immediacy of effect, the overlap of data points among phases, and the consistency of data patterns across participants [54,55]. As we have predicted a delayed treatment effect, we will explore the duration of the delay and whether this delayed effect is consistent across participants, instead of testing immediate effects as proposed by Ledford et al [55]. Data patterns will be analyzed in detail in all 3 phases.

##### Randomization Tests

**Figure 2:**
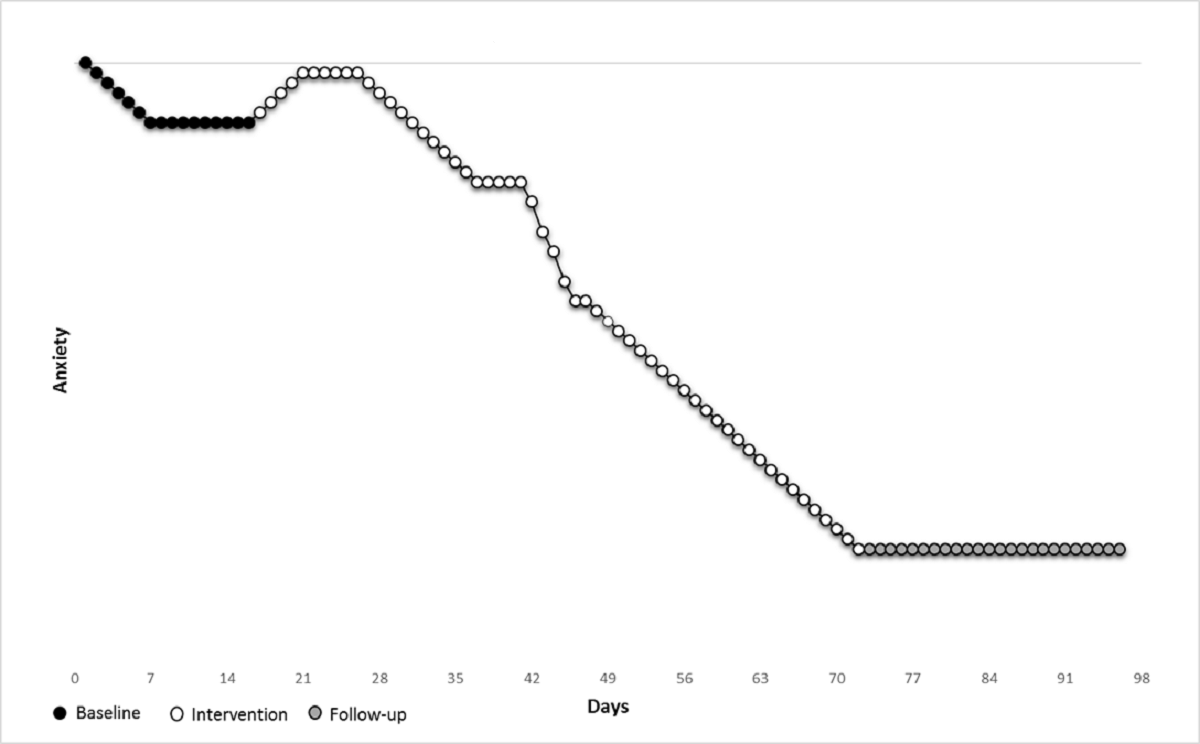
Response function for participants in PROXY.

Randomization tests will be conducted for each participant to test the null hypothesis that PROXY will have no effect on the participants’ anxiety [56-59]. A randomization test for a randomized single-case phase design uses the data in the order they were obtained and is based only on the randomly determined moment in time where the intervention is introduced. Two different test statistics will be used, as follows:

1. Primary test statistic: a mean comparison of phase A and BC will be carried out to investigate mean anxiety levels before and after treatment entry (mean A − mean BC = test statistic). As the starting point of phase B (intervention) is randomly determined and the follow-up phase does not include any new intervention methods, phases B and C are combined. As a mean comparison does not take into account the expected gradual and delayed effect of treatment, we also use a secondary test statistic.
2. Secondary test statistic: response functions [60] will be used to test the hypothesis of delayed and gradual reduction in anxiety (see the Aim section). Informed by previous research on internet-delivered treatment and exposure, we hypothesize that (1) assessment will have a positive effect on participants’ anxiety level; (2) there will be an increase in anxiety at the beginning of treatment, followed by a gradual decrease that becomes steeper after exposure is introduced in module 5; and (3) the effect will be permanent throughout follow-up [14,61,62]. The response function predicted for participants in PROXY is illustrated in Figure 2. Importantly, this is not a prediction of exact anxiety scores but a predicted pattern. The absolute distance between the observed response pattern and the predicted response pattern is used as a test statistic.

As the exact course of treatment effect and the exact scores of anxiety are not known a priori, a multiverse approach will be followed exploring different response functions [60]. These different response functions will follow the same overall response pattern but with variations in the timing as well as the gradient of the response. The multiverse approach entails checking the degree to which the predicted response functions agree or converge with the collected data [63].

We will calculate the primary test statistic as well as the secondary test statistic for each possible randomization scenario of the treatment start using the obtained data. The proportion of test statistic values that are as extreme as, or more extreme than, the test statistic of the true treatment starting point is the *P* value of the randomization test [27,57-59]. This procedure is repeated with data from all 5 participants, and *P* values are combined using the additive method formulated by Onghena and Edgington [64].

##### Effect Size Measure

In addition to the randomization tests, effect size measures will be calculated for each participant. An effect size measure is a standardized indicator that can be compared across studies and is not used as a test statistic in this study. Parker et al [65] recommend using the nonoverlap method Tau-*U* for SCED studies. Tau-*U* is particularly good if data show a baseline trend [65,66]. In addition, the effect sizes will be summarized across all 5 participants by calculating common descriptive statistics (median and range).

##### Analysis of Secondary Outcome Measures

The secondary outcome measures will be analyzed using descriptive statistics because data from only 5 participants will be included.

## Results

The recruitment of parents began in June 2022. As of March 2, 2023, a total of 4 parents have been included in the study. Data collection was expected to cease in April 2023. The project was funded in September 2019. Results are expected to be published in 2024.

## Discussion

### Summary

HA by proxy is a novel research area where much still remains to be investigated. Recent studies suggest that parents experiencing this type of anxiety struggle with how to cope with their health-related worries about their child [4]. When a parent directs special attention toward bodily symptoms and demonstrates health worries regarding their child, research suggests negative consequences for the child, such as negative illness perception and maladaptive symptom coping [5,6,67,68]. Together, this underlines the need for the development and testing of more specific treatment options. Therefore, we developed the first systematic treatment program for HA by proxy, named PROXY.

In this study protocol, we described the treatment components of PROXY and how these components were established in the web-based treatment program. It is a collaboratively developed treatment program that involved clinical experts, web developers, and users in the process. PROXY received positive feedback from patients during development, and it seems to be a usable treatment program. Conducting this first testing and having patients receive treatment in the program may shed light on how to further improve the treatment content of PROXY.

The SCED approach was chosen for the first testing of PROXY because it is particularly useful when testing novel treatments for smaller patient groups [22]. It enables us to measure potential effect with a limited number of participants and to investigate treatment courses for each participant.

### Strengths and Limitations

The randomization and replication across participants are important methodological strengths of this SCED study. By using randomization and randomization tests, confounding factors that are time related are statistically controlled for [27-29]. The external validity is strengthened when this procedure is repeated for several participants [69]. This accommodates the criticism against the standard single-case AB phase design of having low internal validity and a lack of multiple phases without data collected concurrently [70,71].

The selected test statistics for the randomization test are mean comparison and predicted response functions. Using test statistics based on predicted response functions for SCED studies is a newly described framework with the purpose of offering statistical analysis for data with nonlinearity and delayed or gradual changes to the primary outcome measure, as is often seen in psychological treatment [60]. The advantage of response functions is the ability to test very specific developmental trajectories—this is central when testing therapeutic interventions where the introduction of therapeutic techniques can be hypothesized to cause sudden and temporary increase or decrease in symptoms (eg, exposing oneself to feared stimuli may initially increase one’s anxiety symptoms). However, the great specificity of the trajectory described by a response function also means that there is a high risk of failing to reject the null hypothesis. Therefore, primary analyses include visual analysis. Furthermore, using the multiverse approach enables the testing of whether slight adaptations of the characteristics of the trajectory are more fitting.

### Importance of This Study

As the treatment for patients with HA by proxy has not been previously examined, this study is particularly important in terms of describing the treatment development and content as well as the research protocol for using a SCED for the first testing of PROXY. Applying a SCED provides us with the opportunity to investigate the effect of the treatment in a small sample and explore individual treatment courses in detail. Furthermore, the detailed visual analysis provides implicit tests of treatment mechanisms. This has relevance in clinical practice and can potentially be used to inform larger studies on the mechanisms of change and when and how the treatment may be offered to patients in the future [22]. In addition, this study may help to further refine the treatment content of PROXY.

## Abbreviations

ACT: acceptance and commitment therapy
ARCS: Adult Response to Children’s Symptoms
CBT: cognitive behavioral therapy
ESQ: Experience of Service Questionnaire
HA: health anxiety
HAPYS: Health Anxiety by Proxy Scale
iACT: internet-delivered acceptance and commitment therapy
PCS-P: Pain Catastrophizing Scale–Parent version
REDCap: Research Electronic Data Capture
SCAN: schedules for clinical assessment in neuropsychiatry
SCED: single-case experimental design
SCL: Symptom Checklist
WHO-5: World Health Organization-5 Well-Being Index

## Data Availability

Data sharing is not applicable as this is a protocol paper where no data sets were generated or analyzed.
